# Two-Dimensional Sn_2_Te_6_As_2_ as a Promising Thermoelectric Material: Insights from Density Functional and Boltzmann Transport Theories

**DOI:** 10.1021/acsomega.6c03274

**Published:** 2026-07-13

**Authors:** Isaac M. Felix, Daniel C. Café, Laerte P. de Melo, Fábio Lúcio L. de Mendonça, Raphael M. Tromer, Marcelo L. Pereira

**Affiliations:** † Center for Agri-food Science and Technology, Federal University of Campina Grande, Rua Jairo Vieira Feitosa 1770, 58840-000 Pombal, Paraíba, Brazil; ‡ College of Technology, Department of Electrical Engineering, Campus Universitário Darcy Ribeiro, University of Brasília, 70910-900 Brasília, Federal District, Brazil; § College of Technology, Department of Electrical Engineering, Professional Postgraduate Program in Electrical Engineering, Campus Universitário Darcy Ribeiro, University of Brasília, 70910-900 Brasília, Federal District, Brazil; ∥ Institute of Physics, Campus Universitário Darcy Ribeiro, University of Brasília, 70910-900 Brasília, Federal District, Brazil

## Abstract

The efficient conversion of waste heat into electricity is a key challenge in energy sustainability, driving the search for novel thermoelectric materials with enhanced performance. In this work, we investigate the thermoelectric properties of the two-dimensional (2D) material Sn_2_Te_6_As_2_, a theoretically predicted compound identified through machine-learning-assisted materials discovery, using first-principles calculations combined with Boltzmann transport theory. Our results reveal an exceptionally low lattice thermal conductivity of κ^latt^ = 0.24 W/mK at room temperature, which is crucial in improving thermoelectric efficiency by limiting heat dissipation. Additionally, the electronic transport properties, evaluated under the relaxation time approximation (RTA), confirm the isotropic nature of charge transport, ensuring balanced electrical conduction in different crystallographic directions. The figure of merit (ZT) reaches 0.69 for holes and 0.61 for electrons at 300 K, increasing to a peak of 0.80 and 0.79, respectively, at 600 K. The combination of ultralow lattice thermal conductivity, favorable charge transport characteristics, and an optimal operating temperature range suggests that Sn_2_Te_6_As_2_ could be an effective thermoelectric material. These properties make it particularly promising for applications in waste heat recovery and midtemperature energy conversion. These findings offer valuable insights into the fundamental properties of this material and establish a solid foundation for future theoretical and experimental studies aimed at optimizing its thermoelectric performance.

## Graphical abstract

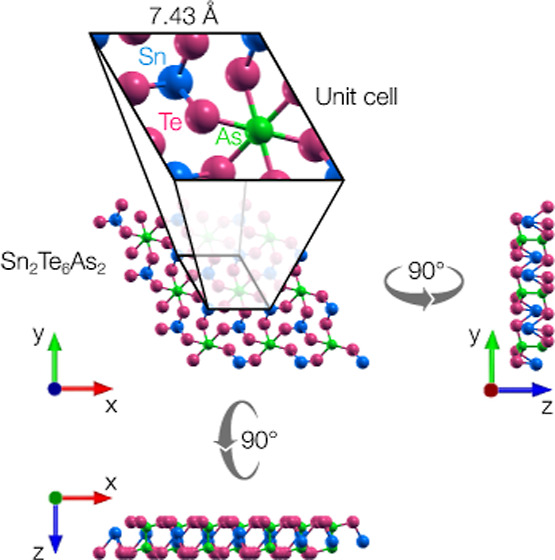

## Introduction

The conversion of waste heat into electricity through the thermoelectric effect has been extensively studied to enhance energy efficiency in electronic devices, industrial systems, and renewable energy applications.^1,2^ In this context, the performance of a thermoelectric material is quantified by the figure of merit (ZT), defined as

1 $$ZT=\frac{\sigma S^{2}T}{\kappa^{elec}+\kappa^{latt}}$$

where σ is the electrical conductivity, *S* is the Seebeck coefficient, *T* is the absolute temperature, and κ^elec^ and κ^latt^ are the electronic and lattice contributions to the thermal conductivity, respectively.^3^ For effective thermoelectric applications, an ideal material should exhibit a high power factor (σ*S* ^2^) and low lattice thermal conductivity, minimizing parasitic heat dissipation while maintaining efficient energy conversion.^4^

Among the materials investigated for thermoelectric applications, two-dimensional (2D) nanomaterials have emerged as promising candidates due to their high surface-to-volume ratio^5^ and, in some cases, a combination of favorable electronic properties and reduced lattice thermal conductivity.^6,7^ Unlike their three-dimensional (3D) counterparts, 2D materials frequently exhibit modified phonon dispersion, resulting in strong phonon scattering and reduced thermal transport.^8,9^ Moreover, band structure engineering in these materials allows optimization of the Seebeck coefficient and power factor, thereby enabling high ZT values.^10^ Notable examples include doped graphene,^11,12^ transition metal dichalcogenides (TMDs),^13,14^ telluride,^15^ and phosphorene-based,^16^ which have been extensively studied for thermoelectric and electronic applications.

Among 2D materials, chalcogenides containing tin (Sn), tellurium (Te), and arsenic (As) have demonstrated significant thermoelectric potential.^17^ Fang et al. analyzed the thermoelectric properties of SnX monolayers (X = O, S, Se), revealing low lattice thermal conductivity and high ZT values, reaching up to approximately 1.40 in SnSe.^18^ Similarly, Gupta and colleagues investigated the SnS monolayer. They identified a distorted NaCl-type structure as the most stable configuration, exhibiting a ZT of 1.36 at 300 K and 5.0 at 600 K, significantly surpassing its bulk counterpart.^19^ Additionally, tellurides of group XIV elements, such as XTe (X = Ge, Sn, Pb), have been widely explored. Dingbo Zhang and coauthors identified ZT values exceeding 1.50 at 900 K and notably low lattice thermal conductivities, such as 1.30 W/mK for GeTe, 3.60 W/mK for SnTe, and 4.30 W/mK for PbTe, at 300 K.^20^ Furthermore, Xu et al. examined the Sb_2_Te_3_ monolayer, identifying a reduced lattice thermal conductivity of 0.72 W/mK at 400 K and a maximum ZT of 0.78 at 800 K, establishing this material as a relevant candidate for thermoelectric applications.^20^

In addition to tellurides, materials composed of group VA elements (As, Sb, and Bi) have also exhibited tunable electronic properties, favoring thermoelectric applications. Dong-Chen Zhang et al. investigated β-As, -Sb, and -Bi monolayers, reporting a substantial reduction in lattice thermal conductivity from As to Bi, with values decreasing from 8.95 W/mK for β-As to only 0.89 W/mK at 300 K.^21^ Furthermore, their study estimated maximum *ZT* values of approximately 0.40, 0.62, and 0.60 for As, Sb, and Bi monolayers under optimal n-type doping conditions. Notably, depending on the doping level and the approach used to estimate the electronic thermal conductivity, the *ZT* values can be further enhanced.^21^ While the literature presents a broad range of thermoelectric materials containing Sn, Te, and As separately, no investigations have explored their combination within a single compound.

Advancements in computational material discovery techniques have driven the search for new 2D thermoelectric materials. Recently, Lyngby and Thygesen proposed a deep generative model, the Crystal Diffusion Variational Autoencoder (CDVAE), to predict stable 2D structures.^22^ Trained on a data set of 2615 2D materials, this model generated over 10,000 new structures, of which 8599 exhibited formation energies below 0.3 eV/atom above the convex hull (Δ*H*_hull_), suggesting potential thermodynamic stability. Among these, 2004 materials were identified with Δ*H*_hull_ < 50 meV/atom, making them promising candidates for further theoretical investigations.^22^ One such material, Sn_2_Te_6_As_2_, integrates tin, tellurium, and arsenic into a novel 2D structure, exhibiting a heat of formation of −0.04 eV/atom and Δ*H*_hull_ = 0.08 eV/atom, suggesting good thermal stability. This material was subsequently incorporated into the Computational 2D Materials Database (C2DB), where its crystal structure and thermodynamic stability were characterized.^23^

In this work, we investigate the thermoelectric properties of Sn_2_Te_6_As_2_ using first-principles calculations based on Density Functional Theory (DFT) and Boltzmann transport theory. We analyze its electronic structure, charge transport properties, and lattice thermal conductivity, assessing its potential for thermoelectric applications. Our results indicate that Sn_2_Te_6_As_2_ exhibits a low lattice thermal conductivity of 0.24 W/mK, promoting high thermoelectric performance. The figure of merit reaches a maximum value of 0.80 at 600 K, positioning this material as a promising candidate for waste heat recovery and midtemperature thermoelectric energy conversion.

### Methodology

The structural, electronic, and thermoelectric properties of Sn_2_Te_6_As_2_ were investigated using first-principles calculations based on DFT. All computations were performed within the framework of the plane-wave pseudopotential method as implemented in the Quantum ESPRESSO (QE) package.^24^ Exchange–correlation effects were treated within the Generalized Gradient Approximation (GGA), adopting the Perdew–Burke–Ernzerhof (PBE) functional.^25^

Structural optimizations were conducted to determine the most stable atomic configuration, enforcing convergence criteria such that residual atomic forces were reduced below 0.01 eV/Å. A vacuum spacing of approximately 20 Å was introduced along the out-of-plane (*z*) direction to suppress spurious interactions induced by periodic boundary conditions. Electronic structure calculations, including band dispersion and density of states (DOS), were carried out using a Monkhorst–Pack *k*-point mesh with a resolution of 20 × 20 × 1, ensuring accurate sampling of the Brillouin zone.

Charge transport properties were evaluated within the relaxation time approximation (RTA), assuming an isotropic transport regime where electronic bands remain unperturbed by temperature and doping effects. The key thermoelectric coefficients-including σ, *S*, and κ^elec^, were computed using the BoltzTraP code,^26^ which employs the semiclassical Boltzmann transport formalism under the rigid band approximation.

The temperature and chemical potential dependence of these transport coefficients were obtained through integration of the energy-resolved conductivity tensor σ_*ij*_(ϵ), weighted by the derivative of the Fermi–Dirac distribution function *f*_0_. The transport coefficients are defined as follows

2 $$\sigma_{ij}\left(T,\mu \right)=\frac{1}{\Omega }\int {\mathrm{\sigma ̅}}_{ij}\left(\epsilon \right)\left[\frac{-\partial f_{0}\left(T,\epsilon ,\mu \right)}{\partial \epsilon }\right]d\epsilon$$

3 $$\begin{array}{l} S_{ij}\left(T,\mu \right)=\frac{1}{eT\Omega \sigma_{ij}\left(T,\mu \right)}\int {\mathrm{\sigma ̅}}_{ij}\left(\epsilon \right)\left(\epsilon -\mu \right)\\ \times \left[\frac{-\partial f_{0}\left(T,\epsilon ,\mu \right)}{\partial \epsilon }\right]d\epsilon \end{array}$$

and

4 $$\begin{array}{l} \kappa_{ij}^{elec}\left(T,\mu \right)=\frac{1}{e^{2}T\Omega }\int {\mathrm{\sigma ̅}}_{ij}\left(\epsilon \right){\left(\epsilon -\mu \right)}^{2}\\ \times \left[\frac{\partial f_{0}\left(T,\epsilon ,\mu \right)}{\partial \epsilon }\right]d\epsilon \end{array}$$

where Ω denotes the unit cell volume, *e* is the elementary charge, and indices *i*, *j* refer to Cartesian components. Within the RTA approach, both σ_*ij*_(*T*, μ) and κ_*ij*_^elec^(*T*,μ) were computed under the assumption of a constant relaxation time (τ).

The κ^latt^ was determined following previously established methodologies by our research group,^27,28^ ensuring consistency in the evaluation of phonon transport mechanisms. The thermoelectric performance of Sn_2_Te_6_As_2_ was characterized by the dimensionless figure of merit, given in eq 1.

## Results and Discuss

To explore the thermoelectric properties of 2D materials, we selected a promising candidate from the C2DB based on its favorable ZT and structural characteristics. Materials composed of Sn and Te with buckled atomic arrangements have been identified as excellent candidates for thermoelectric applications due to their inherently low lattice thermal conductivity, which enhances heat confinement and energy conversion efficiency.

The investigated system corresponds to the stoichiometric composition Sn_2_Te_6_As_2_ (ABC_3_), comprising ten atoms per unit cell. This material remains largely unexplored in the literature, making it an interesting subject for theoretical analysis. Figure 1 illustrates the atomic arrangement, where distinct colors represent different chemical species (Sn – blue, Te – pink, and As – green). The system crystallizes in a trigonal unit cell, with optimized in-plane lattice parameters of 7.43 Å and an out-of-plane dimension of approximately 20 Å. The corresponding lattice angles are 90°, 90°, and 120.18°. The As–Te bond length is approximately 2.68 Å, whereas the Sn–Te bonds exhibit an average length of 3.08 Å.

**Figure 1:**
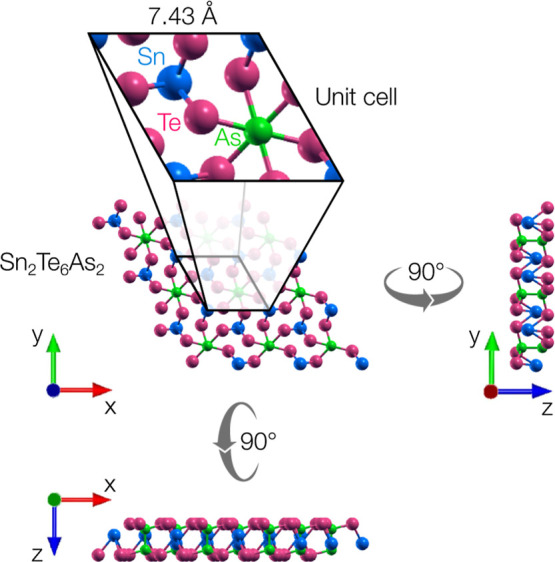
Atomic structure of the Sn_2_Te_6_As_2_ monolayer. The central panel presents the top view of the material, where different atomic species are color-coded: Sn (blue), Te (pink), and As (green). The inset highlights the unit cell, illustrating the atomic coordination environment. Side views, rotated by 90° along different axes, provide additional perspectives of the monolayer, emphasizing its buckled structure and layered arrangement.

The structural optimization reveals that the equilibrium unit cell encompasses a total area of 48.34 Å^2^ and a thickness of 3.76 Å. Figure 1 provides a comprehensive visualization of the material, including a detailed representation of the unit cell and its atomic arrangement. The inset highlights the local coordination environment, illustrating the atomic connectivity of Sn, Te, and As. Additionally, two side views (rotated by 90° along different axes) depict the material’s layered structure, emphasizing its anisotropic nature.

The system belongs to space group P-3 (IT Number 147), indicating its underlying symmetry properties. This material was initially identified within the Lyngby22_LDP data set and is classified as thermodynamically stable.^22^ Its energy above the convex hull is 0.08 eV/atom, which is well within the threshold of stability (typically up to 0.2 eV/atom). Additionally, the cohesive energy (*E*_cohe_) is computed as −4.5 eV/atom, using the expression

5 $$E_{cohe}=\frac{E_{{Sn}_{2}{Te}_{6}{\text{As}}_{2}}-2\left(E_{Sn}+E_{\text{As}}\right)-6E_{Te}}{10}$$

where represents the total energy of the Sn_2_Te_6_As_2_ monolayer, and *E*_Sn_, *E*_As_, and *E*_Te_ correspond to the total energies of isolated Sn, As, and Te atoms, respectively. This result further supports the thermodynamic stability of the material.

To assess potential magnetic effects, we performed spin-polarized calculations. The results indicate that the structure does not exhibit an intrinsic magnetic moment, leading to a net zero magnetization. This absence of magnetization suggests that Sn_2_Te_6_As_2_ is a nonmagnetic system, making it particularly suitable for thermoelectric applications where magnetic interactions could introduce unwanted scattering mechanisms.

The electronic band structure of Sn_2_Te_6_As_2_ was computed along a selected high-symmetry path in the first Brillouin zone, as depicted in Figure 2a. The chosen path includes the following special symmetry points: Γ = (0, 0, 0), *X* = (half, 0, 0), *U* = (one-third, one-third, 0), Γ = (0, 0, 0), *Y* = (0, half, 0), and *U* = (one-third, one-third, 0). These points were selected to ensure a comprehensive representation of the electronic dispersion within the material.

**Figure 2:**
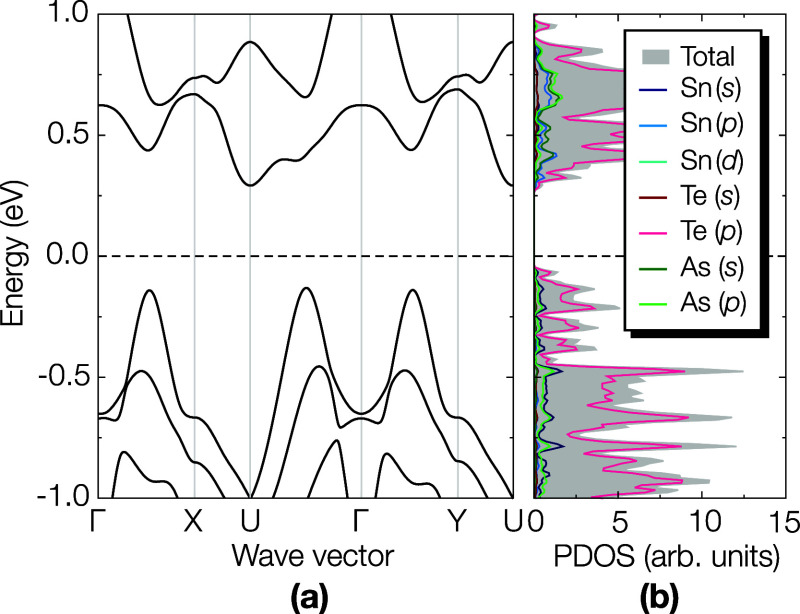
Electronic band structure of Sn_2_Te_6_As_2_ (a) along high-symmetry points in the first Brillouin zone, and projected density of states (b), depicting the contributions of Sn, Te, and As atomic orbitals.

The results indicate that Sn_2_Te_6_As_2_ exhibits semiconducting behavior characterized by an indirect band gap with a PBE value of 0.42 eV. The valence band maximum (VBM) is located along the Γ-X, -U, and -Y paths, while the conduction band minimum (CBM) is positioned at the U point. This spatial separation between the VBM and CBM confirms the indirect nature of the bandgap. Additionally, the curvature of both valence and conduction bands indicates a relatively low effective mass, which is beneficial for charge carrier mobility. The absence of flat bands suggests favorable electronic transport properties, reinforcing the potential of this material for thermoelectric applications.

The projected density of states (PDOS) analysis provides further insights into the orbital contributions to the electronic structure. As shown in Figure 2b, the valence states predominantly comprise the tellurium p orbitals, which exhibit the highest intensity. The arsenic and tin contributions are also present but with lower intensity. Specifically, the *s* states of Sn and the *p* states of As are mainly localized within the valence bands, whereas the p orbitals of both Sn and As contribute more significantly to the conduction bands. The dominance of tellurium states can be attributed to the stoichiometry of the crystal, which consists of three Te atoms for each Sn and As atom, leading to a strong influence of Te on the electronic states.

Another noteworthy observation from the PDOS analysis is the negligible contribution of Sn d orbitals within the energy range considered. While these states play a role in the bonding interactions within the lattice, their impact on the valence and conduction bands near the Fermi level is minimal. This result suggests that the primary electronic transport characteristics of Sn_2_Te_6_As_2_ are dictated by p-type orbitals, which are typically advantageous for thermoelectric performance due to their higher carrier mobility and density of states.

The influence of these electronic properties on transport behavior can be further explored by analyzing temperature-dependent electrical conductivity and relaxation time. Figure 3 presents the variation of electrical conductivity with temperature, with an Arrhenius plot displayed in the inset. Below, the relaxation time as a function of temperature is also shown. As outlined in the methodology, thermoelectric properties (excluding the Seebeck coefficient), such as electrical conductivity and the electronic contribution to thermal conductivity, are typically expressed in units of relaxation time due to the adoption of the constant relaxation time approximation (CRA). Consequently, determining absolute values for these quantities requires an explicit expression for the relaxation time as a function of temperature.

**Figure 3:**
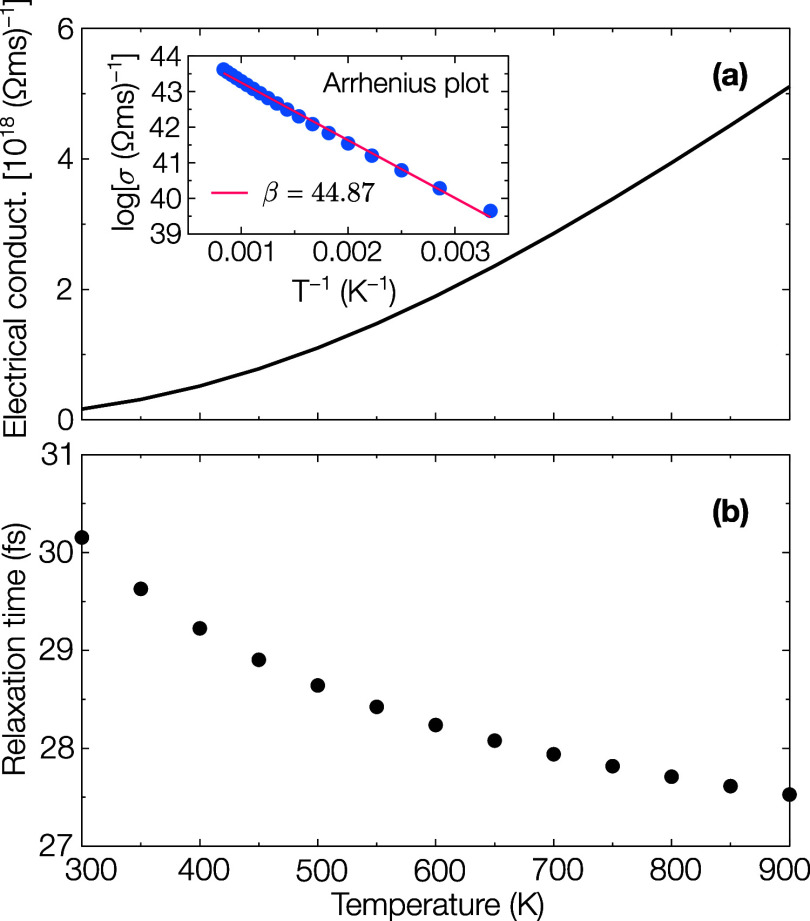
Temperature dependence of electrical conductivity (a) and relaxation time (b) for Sn_2_Te_6_As_2_. The inset in (a) shows the Arrhenius plot.

To obtain this expression, we employ the methodology previously proposed by our research group,^27^ which estimates the relaxation time based on a reference system through a fitting procedure. Initially, the electrical conductivity is evaluated as a function of temperature under the assumption of zero chemical potential, representing a scenario without excess charge carriers, as depicted in Figure 3. From this, an Arrhenius plot is constructed, where the linear coefficient extracted from the logarithmic representation provides the parameter β, which is used in the fitting procedure. The relaxation time is then determined through the following expression

6 $$\tau \left(T\right)=\frac{\Gamma \left(T_{0}\right)}{\Gamma \left(T\right)}\frac{\log\left(\Gamma \left(T_{0}\right)\right)}{\log\left(\Gamma^{Ref}\left(T_{0}\right)\right)}\tau^{Ref}\left(T_{0}\right)$$

where *T*_0_ is the reference temperature, τ^ref^ corresponds to the relaxation time of a similar system, and Γ­(*T*) is an auxiliary function given by

7 $$\Gamma \left(T\right)=\log\left(\frac{\sigma \left(T\right)}{\beta }\right)$$

Applying this procedure, with the estimated linear coefficient β = 44.87 and adopting τ(300) = 29.40 fs from the SnSe system,^29^ the auxiliary function at the reference temperature was determined as Γ^ref^(300) = 32.79. These parameters yield the temperature-dependent relaxation time for Sn_2_Te_6_As_2_, as shown in the lower graph of Figure 3. The results exhibit a characteristic decay with increasing temperature, reflecting the intensification of scattering mechanisms at elevated thermal conditions. As phonon–phonon and electron–phonon interactions become more prominent at higher temperatures, increased collision events contribute to a progressive reduction in relaxation time.

For the Sn_2_Te_6_As_2_ system, the relaxation time at room temperature is slightly above 30 fs, gradually decreasing to approximately 27.5 fs at high temperatures (*T* = 900 K). Additionally, the thermoelectric coefficients were computed using an averaged transport tensor, considering the isotropic nature of the system, which exhibits negligible differences along the *x* and *y* crystallographic directions.

With the relaxation time established as a function of temperature, it becomes possible to compute absolute values for key transport coefficients, including electrical conductivity, the electronic contribution to thermal conductivity, the Seebeck coefficient, and the power factor. The electronic structure directly influences these properties, particularly the distribution of states around the Fermi level. As the band structure analysis reveals, the Fermi level is positioned asymmetrically relative to the valence and conduction bands, influencing charge carrier dynamics and the overall thermoelectric response. This asymmetry is crucial in determining transport behavior, as it governs electronic conduction and thermal transport mechanisms.

Having established the foundational aspects of carrier dynamics, we now analyze temperature-dependent transport properties. Figure 4 presents the evolution of electrical conductivity, electronic thermal conductivity, Seebeck coefficient, and power factor as chemical potential functions. Negative values correspond to hole transport, while positive values indicate electron transport. The electrical conductivity, shown in Figure 4a, exhibits distinct peak values for both charge carriers. The highest conductivity for electrons is observed at μ = 0.50 eV, reaching 0.90×10^6^ (Ω m)^−1^, whereas for holes, the maximum occurs at μ = −0.55 eV, with a peak value of 2.35×10^6^ (Ω m)^−1^. The influence of temperature is evident, as increasing thermal energy enhances carrier scattering, leading to a reduction in conductivity at higher temperatures.

**Figure 4:**
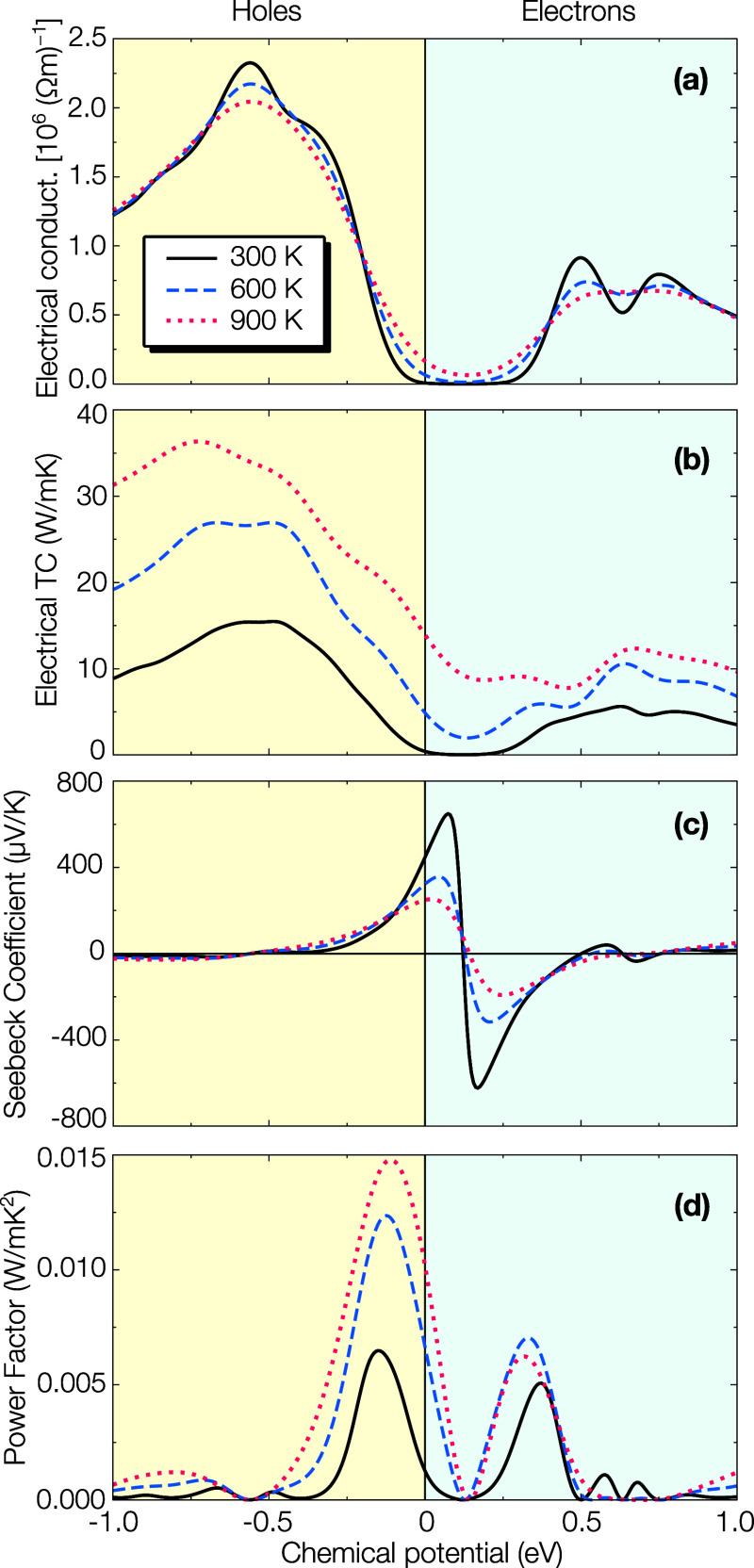
Transport properties of Sn_2_Te_6_As_2_ as a function of chemical potential at different temperatures. Electrical conductivity (a), electronic contribution to thermal conductivity (b), Seebeck coefficient (c), and power factor (d). Negative chemical potentials correspond to hole transport (shaded in yellow), while positive values correspond to electron transport (shaded in blue).

The electronic contribution to thermal conductivity, shown in Figure 4b, follows a distinct trend, increasing monotonically with temperature. Unlike electrical conductivity, no saturation effect is observed within the analyzed range. The peak value of κ^elec^ occurs at μ = −0.50 eV, reaching 15.0 W/mK. This trend highlights how enhanced carrier activity contributes to heat transport via electronic mechanisms.

The Seebeck coefficient further characterizes the thermoelectric response, presented in Figure 4c. This parameter quantifies the voltage induced by a temperature gradient and provides crucial insights into thermoelectric conversion efficiency. The maximum absolute value of 620.0 μV/K is observed at μ = 0.15 eV, with an evident asymmetry between electron and hole contributions. The positioning of these peaks suggests that electron transport is slightly more favorable, reinforcing the role of asymmetry in optimizing thermoelectric performance.

To assess the overall efficiency of charge carrier-based energy conversion, the power factor (σ*S* ^2^) is shown in Figure 4d. The highest value is achieved at μ = −0.15 eV, reaching 0.006 W/mK^2^. The power factor improves significantly with increasing temperature, further supporting the material’s suitability for high-temperature thermoelectric applications. Additionally, the results confirm that transport behavior remains isotropic, as the computed transport coefficients exhibit negligible variation along the x and y crystallographic directions. While individual tensor components were analyzed separately, their near-identical values justify presenting the averaged results, reinforcing the isotropic nature of transport in Sn_2_Te_6_As_2_.

To quantify the material’s thermoelectric efficiency through the figure of merit ZT, we first determine the lattice thermal conductivity in addition to the transport coefficients previously discussed. The computation of κ^latt^ was performed using the approximation developed in reference,^28^ which introduces an analytical expression for estimating the lattice thermal conductivity at room temperature.

8 $$\kappa^{latt}\left(300\right)=\frac{\mathrm{\nu ̅}\times E_{VIB}}{L\times \delta }$$

where ν̅ represents the average phonon frequency, *L* is the lattice parameter, *E*_VIB_ corresponds to the slope coefficient obtained from the Arrhenius plot, and δ is a free parameter calibrated based on a structurally similar reference system. All calculations were conducted using the MOPAC16 software,^30^ which employs a semiempirical approach within the PM7 parametrization scheme.^31^

The parameter δ was calibrated using SnSe as a reference material, the system previously employed for determining the relaxation time. By fitting the model to reproduce the experimentally reported lattice thermal conductivity of SnSe (2.25 W/mK), the optimal calibration yielded δ = 7 K. Applying this parameter to Sn_2_Te_6_As_2_, we obtained an average lattice thermal conductivity of κ^latt^ = 0.24 W/mK, highlighting the strong phonon scattering mechanisms in this system.

The temperature-dependent behavior of κ^latt^ follows the typical scaling law observed in two-dimensional materials, exhibiting a power-law decay proportional to *T* ^–1^. Figure 5 illustrates the variation of lattice thermal conductivity as a function of temperature. At the same time, the inset presents the Arrhenius plot used to extract the vibrational parameter *E*_VIB_. The observed trend confirms the suppression of thermal transport at higher temperatures, consistent with enhanced phonon–phonon scattering interactions.

**Figure 5:**
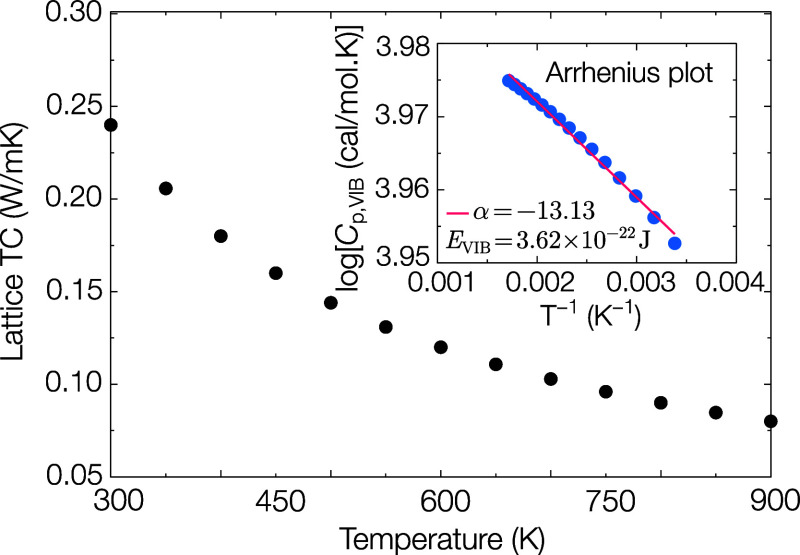
Temperature dependence of the lattice thermal conductivity of Sn_2_Te_6_As_2_. The inset shows the Arrhenius plot used to determine the vibrational energy parameter *E*_VIB_.

With all the relevant transport coefficients determined, we now compute the dimensionless figure of merit ZT, as defined in eq 1. Figure 6 presents the variation of ZT as a function of chemical potential in the range of −2.0 to 2.0 eV, considering three temperature conditions: 300, 600, and 900 K. The results reveal two prominent peaks near the Fermi level, corresponding to hole and electron transport. As previously discussed, the asymmetry in the density of states leads to an uneven distribution of thermoelectric performance, with the peak associated with hole transport occurring closer to the Fermi level. This asymmetry suggests that a lower carrier concentration is required to achieve optimal thermoelectric performance for holes compared to electrons.

**Figure 6:**
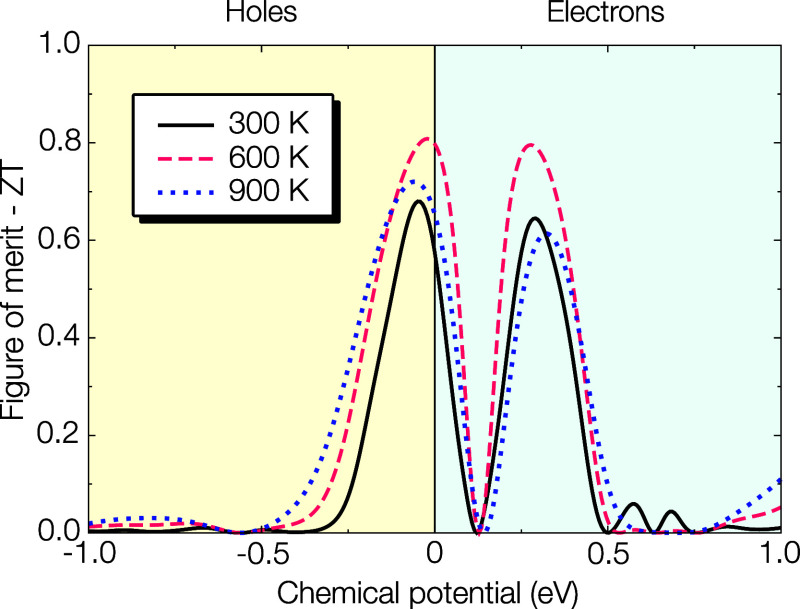
Figure of merit of Sn_2_Te_6_As_2_ as a function of chemical potential for different temperatures. The shaded regions indicate the dominance of hole (left) and electron (right) transport contributions.

The maximum ZT for electrons is observed at approximately 0.25 eV, reaching a peak value of 0.61, whereas the highest ZT for holes occurs around −0.05 eV, attaining 0.69. Furthermore, the maximum ZT evolution with temperature exhibits distinct behaviors for electrons and holes. For electrons, the peak values are 0.61, 0.79, and 0.59 at *T* = 300, 600, and 900 K, respectively. In contrast, the corresponding maximum values for holes are 0.69, 0.80, and 0.71 for the same temperature conditions. In both cases, the ZT values reach their highest efficiency around *T* = 600 K, after which a saturation effect is observed, followed by a slight decline at higher temperatures.

The temperature dependence of ZT reveals a distinct asymmetry between electron and hole transport. Notably, at *T* = 600 K the ZT value surpasses that at *T* = 300 K for both carriers. At *T* = 900 K the trends diverge. The electron ZT (0.59) falls below its 300 K value (0.61), whereas the hole ZT (0.71) remains slightly above it (0.69). This behavior arises from the asymmetry of the Fermi level, which facilitates hole injection more effectively than electron injection. Furthermore, the observed saturation of ZT around *T* = 600 K suggests the onset of a bipolar conduction, where carriers thermally excited across the band gap increasingly contribute to transport properties. This transition is further corroborated by the DOS map presented in Figure 7, which illustrates the variation of electronic states as a function of temperature and chemical potential.

**Figure 7:**
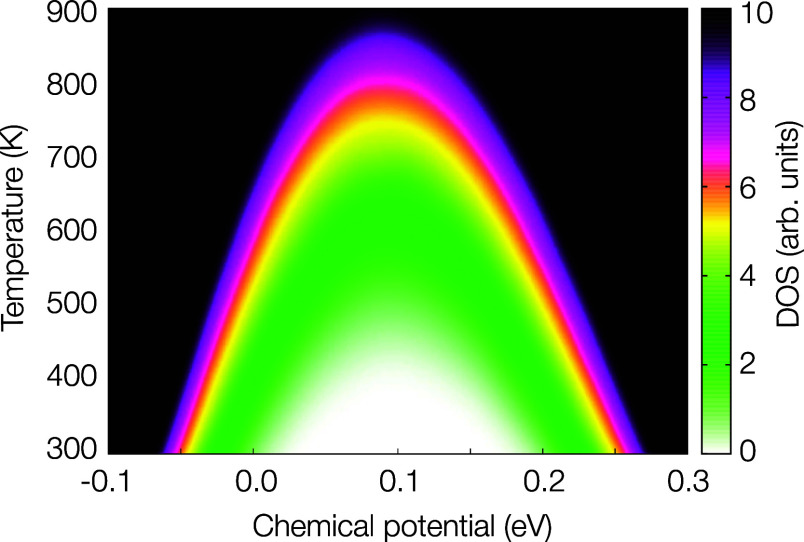
Density of states map of Sn_2_Te_6_As_2_ as a function of temperature and chemical potential.

In Figure 7, lighter regions indicate lower DOS values, characteristic of semiconducting behavior, whereas darker (black) regions correspond to higher DOS values, indicative of metallic character. By examining the DOS at zero chemical potential, corresponding to the Fermi level, we observe a significant increase in electronic states only for temperatures approaching *T* = 600 K. This suggests that, beyond this temperature, bipolar conduction becomes significant, limiting its efficiency as a thermoelectric material. This observation aligns with the saturation trend of the thermoelectric parameters, reinforcing that Sn_2_Te_6_As_2_ is most effective within the intermediate temperature range.

Overall, Sn_2_Te_6_As_2_ demonstrates promising thermoelectric performance, with ZT values of 0.69 for holes and 0.61 for electrons at room temperature. The maximum efficiency is achieved at *T* = 600 K, where ZT reaches 0.80 for holes and 0.79 for electrons. These findings highlight the material’s suitability for thermoelectric applications in intermediate-temperature energy conversion, particularly in waste heat recovery, power generation for industrial processes, and thermoelectric cooling. The combination of high thermoelectric performance and an operational temperature range that aligns with practical applications makes Sn_2_Te_6_As_2_ a promising candidate for future thermoelectric device development.

## Conclusions

In this study, we employed first-principles calculations combined with Boltzmann transport theory to investigate the thermoelectric properties of the two-dimensional material Sn_2_Te_6_As_2_. Our results demonstrate that this material exhibits promising thermoelectric performance, characterized by a figure of merit ZT reaching 0.69 for holes and 0.61 for electrons at room temperature. The thermoelectric efficiency reaches its maximum at *T* = 600 K, where ZT attains peak values of 0.80 and 0.79 for holes and electrons, respectively. This enhancement is primarily driven by the exceptionally low lattice thermal conductivity and the asymmetric electronic structure, which preferentially favors hole transport.

The transport analysis confirmed that Sn_2_Te_6_As_2_ exhibits an isotropic charge transport behavior, as evidenced by the negligible variation of thermoelectric coefficients along different crystallographic directions. Additionally, the density of states analysis as a function of temperature revealed that bipolar conduction sets in at approximately *T* = 600 K. Beyond this temperature, the increasing carrier density leads to a decline in ZT, indicating that the material’s thermoelectric efficiency is optimal within an intermediate temperature range.

Given these findings, Sn_2_Te_6_As_2_ emerges as a strong candidate for thermoelectric applications in the midtemperature regime. Potential applications include waste heat recovery, energy harvesting in industrial processes, and thermoelectric cooling technologies. Future experimental studies will be essential to validate these theoretical predictions and further refine the understanding of this material’s thermoelectric behavior. Additional theoretical investigations, including anharmonic phonon effects and advanced electron–phonon scattering mechanisms, could provide deeper insights into its transport properties and potential for integration into practical thermoelectric devices.
